# Epilepsy Treated by Sulphonal

**Published:** 1891-12

**Authors:** Gilbert A. Bannatyne

**Affiliations:** Late Resident Medical Officer Smithston Asylum and Poorhouse


### EPILEPSY TREATED BY SULPHONAL.

BY

GILBERT A. BANNATYNE, M.D., C.M. Glasg.,

*Late Resident Medical Officer Smithston Asylum and Poorhouse.*

On reading some early notes[1] on "Sulphonal," I came to the conclusion that the drug might prove of great use in certain cases of epilepsy, especially in those which had not been much benefited by the bromides.

In epilepsy we have to treat both cause and effect; for if the fits have been numerous, and if the cause, which

[1] *British Medical Journal*, Vol. I., 1888, p. 864, and Vol. II., 1888, p. 31.

may have lasted for a long time, has disappeared, they may persist because the brain, so to speak, has become used to being epileptic. It has acquired a considerable hyper-excitability, and the fits may have determined secondary lesions, which are often incurable.

Sulphonal can, in suppressing the fits, prevent these secondary lesions, if given in time, and it thus aids in the ultimate cure. Also, when the cause is unknown or inaccessible to treatment, it can still, in lessening the number or severity of the fits, make life more comfortable both to the patient and his friends.

In the following few cases, sulphonal was given at bedtime, in two separate doses. The total quantity given varied from 10 to 40 grains, according to the severity of the symptoms and tolerance of the drug. If pushed too far, it causes the patient to feel sleepy and languid during the day. This should, I think, be avoided. In all my cases, I have found that the fits have either ceased as long as the drug was taken, or have been greatly diminished both in number and intensity. Of course the cases chosen were principally those on whom the more ordinary treatment had little or no effect.

*Case I.*—A. D., æt. 22. Patient had suffered from epilepsy since he was eleven years of age.

Cause was supposed to be a fright. No hereditary history.

The fits began with a cry, and the patient fell foaming at the mouth. He passed his urine without knowing it, during the fits. He was restless at night, cried out every now and then, and slept little. He had about three fits a week. All the ordinary drugs had been tried, but none of them produced any permanent good. Chloral quieted him for the time being, but left him enervated, and the fits which followed were very violent. He is almost a complete idiot. He says he is afraid of someone, who he says runs after him, and that the other patients annoy him. When he speaks he stutters over certain syllables.

The right frontal region is not so prominent as the left. Right pupil slightly dilated.

*Treatment.*—July 1st. Sulphonal, grs. xl., was given at bedtime, in two doses. The patient slept all night, and did not present next day any symptoms of interest. No fits.

July 2nd. Slept all night, after getting sulphonal, grs. xxx. No fits.

July 3rd. No sulphonal.

July 4th. Towards noon, one fit. Was given sulphonal, grs. xl., at bedtime.

July 5th. No sulphonal. One fit about 8 p.m.

July 6th, 7th, and 8th. Got sulphonal, grs. xxx., each night. No fits.

July 9th. Sulphonal, grs. xx. No fits.

July 10th, 11th, and 12th. Sulphonal, grs. xv., given each night. No fits.

July 13th and 14th. No sulphonal. One fit towards the evening of the 14th.

July 15th, 16th, 17th, and 18th. Sulphonal, in doses ranging from 40 grains to 10. No fits.

From the 18th July the patient received regularly a dose of sulphonal at bedtime, varying in quantity according to his state. It was found that his fits could be kept in check almost entirely by this means, and his general health has much improved.

*Case II.*—H. B., æt. 12. Has had fits, for the last year, almost daily.

No cause can be given, and her family history is good.

The fits come on without aura. After the fits she has severe frontal and occipital headache, and is very difficult to manage. After undergoing a course of the bromides, she was put on sulphonal; 20 grains were given for a fortnight, when the dose was reduced to 10 grains. At the end of a month it was stopped. During this period she had fits on the first three days of treatment, but has had none since; and now appears perfectly well, one year after her discharge.

*Case III.*—L. B., æt. 24, farm labourer. He has suffered from fits since he was ten years of age. Cause unknown, but he comes of a nervous family.

The fits occur almost daily; and he is noisy at night, and very difficult to manage, being destructive and pugnacious. Chloral had been tried with fair success to quiet him, but had no effect on the fits. He was put on sulphonal, getting it regularly at bedtime, in doses of 20 to 10 grains. Since this has been carried out, the fits, although not ceasing, have diminished in number and severity. He is much more easily managed, and does not fight so often. His weight has increased, and his general health is much improved.

*Case IV.*—C. H., æt. 40, farm labourer. He has had fits since 18 years of age. No cause known. Family history good. The fits occur about once a week. They are followed by prolonged headaches and vertigo. His mind is much enfeebled, and he is said to be a thoroughly bad character. His body is covered with bruises and old cicatrices.

For a month he was put on sulphonal, 20 or 10 grains, according to the effect. It was always reduced on the slightest sign of sleepiness during the day. During this period he had only one fit, and did not complain of headache or vertigo after it. Unfortunately, after this period he was removed by his friends, and I have been unable to obtain his after history.

*Case V.*—C. D., æt. 23. Has suffered from fits since he was sixteen years of age. His father and mother died of phthisis, and he has a brother who is epileptic.

The fits occur once or twice a month. About an hour before a fit he feels sick. The right side is the one principally affected. Left frontal region much less prominent than right. For ten days he got sulphonal, grs. xv.; but then, as it produced great lassitude and desire to sleep, the dose was reduced to grs. v. This was continued for a month, and as he had no return of the fits he was discharged well, and I hear has remained so ever since (over a twelvemonth).

*Case VI.*—A. C., æt. 53. Has suffered from epilepsy for the last five years. Nothing could be made out of the family history. Five years ago he had an attack of right hemiplegia.

At the present time his intelligence and memory are feeble. His right forearm is flexed to a right angle with the upper arm, and the fingers are bent inwards until they rest on the epigastrium; the joints are quite stiff, and the muscles are much atrophied. The fits occur almost nightly. He is very dirty and wet.

Sulphonal, grs. x. to grs. xx., was given for a month, and during this period he had only four fits and three attacks of vertigo. At the end of the month he was put on bromide of potassium as an experiment; but the fits recurred more frequently, and he was again placed on small doses of sulphonal. He gets it daily, and continues to improve, and remains fairly free from fits four months after starting the sulphonal treatment.

*Case VII.*—L. J., æt. 17. He has suffered from epilepsy since he was fourteen years of age. Cause unknown. He has a brother who is likewise epileptic.

The fits occur about once a week, and are severe in character. His mind is much enfeebled, and he is greatly given to masturbation. He was put on sulphonal, grs. xv., and this was continued for a month, during which time he had only one fit. He now gets 10 grains every second night, and this keeps him comparatively free from fits. His mental condition remains much the same.

*Case VIII.*—H. D., æt. 33. Has had fits since he was eighteen. An aunt died during an epileptic fit, and he has an uncle who also suffers from epilepsy.

He suffers from slight divergent strabismus, said to have been the result of "infantile paralysis." He has a fit about every two days. Mentally, he is much enfeebled, and is quarrelsome. During one month he got sulphonal, grs. xv., and, as the fits were not so frequent, the dose was reduced to 10 grains every second night. He is now (six months after the commencement of the treatment) much improved both mentally and bodily, and the fits have almost ceased. He is in a fair way, I think, to recover almost completely.

